# Metabolomics Analysis Reveals Specific Novel Tetrapeptide and Potential Anti-Inflammatory Metabolites in Pathogenic *Aspergillus* species

**DOI:** 10.3390/ijms160613850

**Published:** 2015-06-17

**Authors:** Kim-Chung Lee, Emily W. T. Tam, Ka-Ching Lo, Alan K. L. Tsang, Candy C. Y. Lau, Kelvin K. W. To, Jasper F. W. Chan, Ching-Wan Lam, Kwok-Yung Yuen, Susanna K. P. Lau, Patrick C. Y. Woo

**Affiliations:** 1 Department of Microbiology, The University of Hong Kong, Pokfulam, Hong Kong; E-Mails: lee1983@hku.hk (Ki.-C.L.); emily.wt@gmail.com (E.W.T.T.); lkc3309@gmail.com (Ka.-C.L.); h0365593@graduate.hku.hk (A.K.L.T.); candylaucy@gmail.com (C.C.Y.L.); kelvinto@hku.hk (K.K.W.T.); jfwchan@hku.hk (J.F.W.C.); kyyuen@hku.hk (K.-Y.Y.); 2 State Key Laboratory of Emerging Infectious Diseases, The University of Hong Kong, Pokfulam, Hong Kong; 3 Research Centre of Infection and Immunology, The University of Hong Kong, Pokfulam, Hong Kong; 4 Carol Yu Centre for Infection, The University of Hong Kong, Pokfulam, Hong Kong; 5 Department of Pathology, The University of Hong Kong, Pokfulam, Hong Kong; E-Mail: ching-wanlam@pathology.hku.hk

**Keywords:** *Aspergillus*, metabolomics, metabolites, biomarkers, tetrapeptide

## Abstract

Infections related to *Aspergillus* species have emerged to become an important focus in infectious diseases, as a result of the increasing use of immunosuppressive agents and high fatality associated with invasive aspergillosis. However, laboratory diagnosis of *Aspergillus* infections remains difficult. In this study, by comparing the metabolomic profiles of the culture supernatants of 30 strains of six pathogenic *Aspergillus* species (*A. fumigatus*, *A. flavus*, *A. niger*, *A. terreus*, *A. nomius* and *A. tamarii*) and 31 strains of 10 non-*Aspergillus* fungi, eight compounds present in all strains of the six *Aspergillus* species but not in any strain of the non-*Aspergillus* fungi were observed. One of the eight compounds, Leu–Glu–Leu–Glu, is a novel tetrapeptide and represents the first linear tetrapeptide observed in *Aspergillus* species, which we propose to be named aspergitide. Two other closely related *Aspergillus*-specific compounds, hydroxy-(sulfooxy)benzoic acid and (sulfooxy)benzoic acid, may possess anti-inflammatory properties, as 2-(sulfooxy)benzoic acid possesses a structure similar to those of aspirin [2-(acetoxy)benzoic acid] and salicylic acid (2-hydroxybenzoic acid). Further studies to examine the potentials of these *Aspergillus*-specific compounds for laboratory diagnosis of aspergillosis are warranted and further experiments will reveal whether Leu–Glu–Leu–Glu, hydroxy-(sulfooxy)benzoic acid and (sulfooxy)benzoic acid are virulent factors of the pathogenic *Aspergillus* species.

## 1. Introduction

In recent years, infections related to *Aspergillus* species have become an emerging focus of clinical microbiology and infectious disease as the number of patients infected with *Aspergillus* species rose dramatically. In immunocompetent and mildly immunocompromised hosts, *Aspergillus* species rarely cause serious illnesses, except for pulmonary aspergilloma in patients with pre-existing chronic lung diseases and, less commonly, chronic cavitary pulmonary aspergillosis, chronic fibrosing aspergillosis and chronic necrotizing aspergillosis [1,2,3,4,5]. On the other hand, invasive aspergillosis is one of the most important causes of morbidity and mortality in immunocompromised patients, such as those with hematological malignancies undergoing chemotherapy, hematopoietic stem cell and solid organ transplantation and HIV infection [1,6]. Among the known *Aspergillus* species, *Aspergillus fumigatus* is the most common species causing human infections in western countries, whereas *Aspergillus* *flavus* is as important as *A. fumigatus* in our locality and in other Asian countries, and is the second most common *Aspergillus* species associated with human infections in western countries [1,6]. Other *Aspergillus* species commonly associated with human infections include *Aspergillus niger* and *Aspergillus terreus* [1,6].

The successful management of invasive aspergillosis is hampered by difficulties in establishing timely and accurate diagnosis. The gold standard for making a diagnosis is to obtain a positive culture of *A. fumigatus* and to demonstrate histological evidence of mycelial invasion from tissue biopsy. Due to the very sick nature of these patients and often the presence of bleeding diathesis, tissue biopsy is often not possible or acceptable by patients. Although commercial kits for galactomannan antigen detection and our in-house developed Afmp1p and Afmp2p antigen detection assays are available for clinical use, the sensitivities of these tests are far from ideal [2,3,4,5,7]. Molecular tests such as PCR are also used for laboratory diagnosis, but such tests cannot distinguish among environmental contamination, colonization and genuine invasive infection [8,9].

Microbial metabolomics is a relatively new research field that involves the study of the unique chemical fingerprints of the metabolite profiles of microorganisms, and has been used for the characterization of a number of pathogenic microbes [10,11,12,13]. For example, urine metabolomic data can be used for the diagnosis of *Streptococcus pneumoniae* infections and urinary tract infections [14,15,16]. We have also reported the application of metabolomics for identification of mitorubrinol yellow pigment in the pathogenic dimorphic fungus, *Penicillium marneffei*, and characterization of *Tsukamurella* and *Aspergillus* strains [17,18,19], as well as detection of specific metabolites in culture supernatant of *Mycobacterium tuberculosis* which may have important diagnostic applications [20]. Since our previous study showed that metabolomics profiling can be used to distinguish among different *Aspergillus* species [17], we hypothesize that there could be *Aspergillus-*specific novel extracellular metabolites that may be detected in blood and body fluids for laboratory diagnosis of invasive aspergillosis. In order to look for these potential biomarkers of invasive aspergillosis, we characterized the metabolomic profiles of the culture supernatants of *Aspergillus* species and other clinically important fungal species, using ultrahigh performance liquid chromatography-electrospray ionization-quadrupole time-of-flight mass spectrometry (UHPLC–ESI-Q-TOF-MS). Multi- and univariate statistical analyses of the metabolomic profiles were performed to identify *Aspergillus*-specific metabolites.

## 2. Results

### 2.1. Visual Inspection of Total Ion Chromatograms

**Figure 1:**
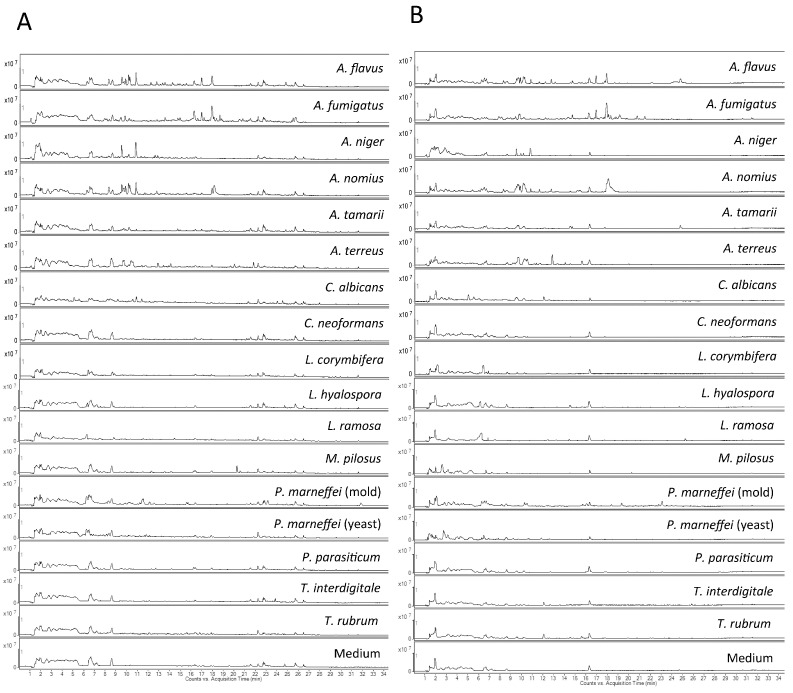
Total ion chromatograms of *Aspergillus* species and other control fungal species used in this study. (**A**) Positive ionization mode; (**B**) Negative ionization mode.

The metabolomes of culture supernatants of the 128 samples, including 30 samples of the 30 *Aspergillus* strains in duplicate and 34 samples from the 31 non-*Aspergillus* fungal strains (six samples were obtained from the mold and yeast forms of the three *P. marneffei* strains) in duplicate, were characterized and compared. UHPLC–ESI-Q-TOF-MS methods, operated in both positive and negative modes, for the analysis of different metabolites in fungal culture supernatant, were developed. Total ion chromatograms (TIC) from the *Aspergillus* strains shared considerable similarity and those of the same *Aspergillus* species showed high similarity, whereas significant differences were observed from the TICs of the different non-*Aspergillus* species. Representative examples of chromatograms from each species obtained in both positive and negative modes are shown in Figure 1A,B, respectively.

### 2.2. Omics-Based Bioinformatics Analysis

There were 32,849 molecular features (MFs) detected in positive electrospray ionization (ESI) mode and 39,303 MFs detected in negative ESI mode. All MFs were subjected to the Mass Profiler Professional (MPP) software for statistical analysis. After filtering by the MPP software, total of 2404 MFs in positive mode and 1737 MFs in negative mode were obtained. Stepwise filtering procedure was used to identify significant MFs in *Aspergillus* strains and to reduce the dimensionality of data prior to principle component analysis (PCA) and partial least squares discriminant analysis (PLS-DA). To compare the metabolomes between *Aspergillus* and non-*Aspergillus* fungal strains, both uni- and multi-variate analyses were performed. Three-hundred-and-six features in positive mode and 44 features in negative mode were further subjected for volcano plot analysis. Using Student’s *t*-test with *p*-value <0.01 and fold-change (*FC*) >16 to compare the *Aspergillus* and non-*Aspergillus* groups, the number of MFs was reduced to 287 features in positive mode and 42 features in negative mode after stepwise filtering for PCA and PLS-DA analyses.

### 2.3. PCA and PLS-DA Modeling

**Figure 2:**
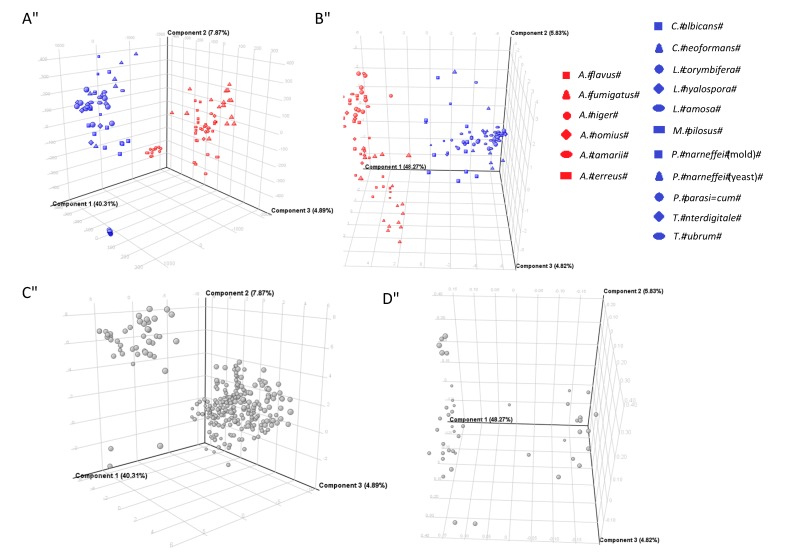
PCA score plots and PCA loading plots of 128 fungal culture supernatant samples. PCA score plots for (**A**) positive ionization mode data and (**B**) negative ionization mode data; PCA loading plots of molecular features for (**C**) positive ionization mode data and (**D**) negative ionization mode data.

To compare the metabolomes between *Aspergillus* and non-*Aspergillus* strains, multivariate analysis was performed. In the positive ionization mode, PCA score plot showed that 48.18% of the total variance in the data was represented by the first two principal components (PCs), in which PC1 explained 40.31% of the variance and PC2 explained 7.87% in the three-dimensional PCA score plots (Figure 2A). In the negative ionization mode, PCA score plot showed that 54.10% of the total variance in the data was represented by the first two PCs, in which PC1 explained 48.27% of the variance and PC2 explained 5.83% in three-dimensional PCA score plots, which gave a better clustering than the positive ionization mode (Figure 2B). All PCA score plots revealed that the *Aspergillus* strains were closely related to each other and can be distinguished from the non-*Aspergillus* strains, based on the first two principal components, where the *Aspergillus* strains were clearly separated from the non-*Aspergillus* strains along PC1. PCA loading plots for the respective MFs, which account for the separation between the *Aspergillus* and non-*Aspergillus* strains, were also generated in either positive ionization mode (Figure 2C) or negative ionization mode (Figure 2D). MFs that showed statistically significance in the *Aspergillus* strains were extracted for further analysis.

**Figure 3:**
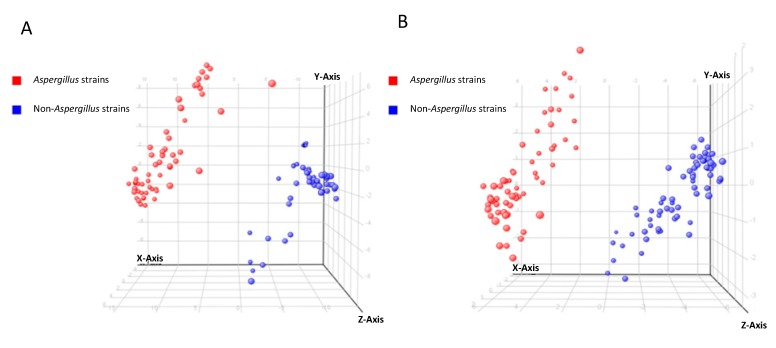
PLS-DA t-score plots of 128 fungal culture supernatant samples. (**A**) Positive ionization mode; (**B**) negative ionization mode.

In view of the significant separation achieved using PCA, supervised PLS-DA using t-score plots was employed to maximize the separation between the *Aspergillus* and non-*Aspergillus* strains, to find potential marker variables, to validate statistical model and to predict sample class membership. In both positive and negative ionization modes, sample classifications using the PLS-DA model achieved 100% accuracy in both recognition and prediction abilities, indicating that the *Aspergillus* and non-*Aspergillus* strains were correctly classified during model training and cross-validation. Excellent separations of the *Aspergillus* and non-*Aspergillus* strains obtained by PLS-DA were shown in Figure 3.

### 2.4. Identification of Potential Biomarkers in Aspergillus Species

**Figure 4:**
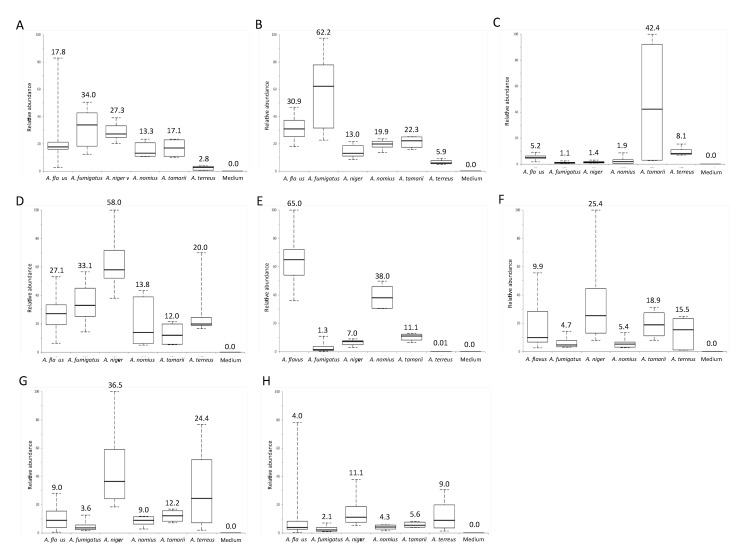
Comparison of abundance of metabolites specific to the six *Aspergillus* species. Box-and-whisker plots of (**A**) 7-methylguanine; (**B**) 1-methylguanine; (**C**) hydroxyl(sulfooxy)benzoic acid; (**D**) (sulfooxy)benzoic acid; (**E**) Leu–Glu–Leu–Glu; (**F**) metabolite with protonated molecular ion of *m*/*z* 292.2645 eluted at 19.55 min; (**G**) metabolite with protonated molecular ion of *m*/*z* 352.2466 eluted at 28.5 min; (**H**) metabolite with protonated molecular ion of *m*/*z* 352.2466 eluted at 28.8 min. Boxes show first to third quartiles; whiskers show 5% and 95% percentiles; middle lines in boxes and numbers on the top of boxes indicate the median of the relative abundance of metabolite.

Potential biomarkers in *Aspergillus* species were identified according to the elution order, MS/MS fragmentation pattern, molecular formula and database search. A total of eight potential biomarkers were identified (Table 1 and Table A1). Box and whisker plots of the integral peak areas for all the eight metabolites were constructed (Figure 4).

**Table 1:** Specific metabolites in culture supernatant of *Aspergillus* species.

Compound	*m*/*z*	Retention Time (min)	Ionization Mode	Ion	MS/MS Fragment Masses	*p* Value ^a^	Molecular Formula	Putative Identity
**1**	166.0724	1.86	Positive	[M + H]+	107.0232, 124.0493, 149.0446, 166.0712	<0.001	C6H7N5O	7-Methylguanine
**2**	166.0724	3.60	Positive	[M + H]+	110.0327, 135.0292, 149.0441, 166.0702	<0.001	C6H7N5O	1-Methylguanine
**3**	232.9775	6.20	Negative	[M − H]−	96.9593, 109.0288, 153.0192, 188.9861, 232.9775	<0.001	C7H6O7S	Hydroxy(sulfooxy)benzoic acid
**4**	216.9822	6.80	Negative	[M − H]−	79.9570, 93.0346, 96.9598, 137.0245, 172.9911, 216.9816	<0.001	C7H6O6S	(sulfooxy)benzoic acid
**5**	503.2749	9.98	Positive	[M + H]+	96.0458, 114.0557, 131.0818, 225.1232, 243.1329, 261.1452, 503.2730	<0.001	C22H38N4O9	Leu–Glu–Leu–Glu
**6**	292.2645	19.55	Positive	[M + H]+	74.0963, 203.1426, 219.1743, 292.2633	<0.001	C19H33NO	No match
**7**	352.2466	28.5	Positive	[M + H]+	81.0189, 94.0269, 165.1127, 194.1155, 210.1103	<0.001	C20H33NO4	No match
**8**	352.2466	28.8	Positive	[M + H]+	81.0191, 94.0268, 165.1126, 194.1156, 210.1105	<0.001	C20H33NO4	No match

^a^ *p* value from ANOVA analysis.

**Figure 5:**
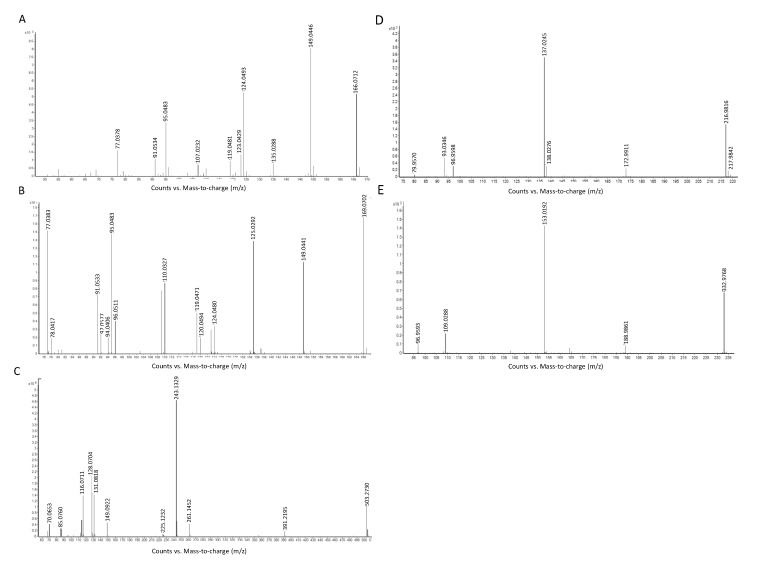
MS/MS spectra of (**A**) 7-methylguanine; (**B**) 1-methylguanine; (**C**) Leu–Glu–Leu–Glu; (**D**) (sulfooxy)benzoic acid and (**E**) hydroxyl(sulfooxy)benzoic acid.

Two metabolites (compounds **1** and **2** in Table 1), at retention time 1.86 and 3.60 min, with the same theoretical [M + H]^+^ of *m*/*z* 166.0724 and molecular formula of C_6_H_7_N_5_O, were identified as 7-methylguanine and 1-methylguanine respectively by database search in METLIN. MS/MS spectra of 7-methylguanine (Figure 5A) and 1-methylguanine (Figure 5B) showed the major fragmentation peak at *m*/*z* 149, which was a result of the loss of an amino group at the C-2 position of the guanine group. Another peak at *m*/*z* 124 in the MS/MS spectra of 7-methylguanine was formed by losing the N-1, N-9 and C-8 position of 7-methylguanine, whereas the peak at *m*/*z* 135 in the MS/MS spectra of 1-methylguanine represent the loss of methyl group at N-1 position and amino group at C-2 position from 1-methylguanine. Among all the six *Aspergillus* species, 7-methylguanine and 1-methylguanine were both expressed in highest abundance in *A. fumigatus* and lowest abundance in *A. terreus.*

The metabolite (compound **5** in Table 1) with [M + H]^+^ of *m*/*z* 503.2749, at a retention time of 9.98 min, and molecular formula of C_22_H_38_N_4_O_9_ matched 24 tetra-peptides by database search in METLIN. The peptide sequence was revealed to be Leu–Glu–Leu–Glu by using Peaks^©^7 (Bioinformatics Solution Inc., Waterloo, ON, Canada). Different peaks in the MS/MS spectrum of the tetra-peptide represented different fragmented peptide ions (*m*/*z* = 96, *b*_1_-H_2_O ion; *m*/*z* = 114, *b*_1_ ion, *m*/*z* = 131, *y*_1_-NH_3_ ion; *m*/*z* = 225, *b*_2_-H_2_O ion; *m*/*z* = 243, *y*_2_-H_2_O and/or *b*_2_ ions and *m*/*z* = 261, *y*_2_ ion) (Figure 5C). Among all the *Aspergillus* species, Leu–Glu–Leu–Glu was expressed in highest abundance in *A. flavus* and lowest abundance in *A. terreus*.

Two metabolites (compounds **3** and **4** in Table 1), with undefined positional isomers, were identified by exact molecular weight, predicted molecular formula, MS/MS fragmentation pattern and literature search. Compound **4** in Table 1, with deprotonated molecular ion at *m*/*z* 216.9822 and at a retention time of 6.80 min, was identified as C_7_H_6_O_6_S. The MS/MS fragmentation pattern (Figure 5D) supported that the chemical structure of compound **4** is (sulfooxy)benzoic acid. The peak at *m*/*z* 173 referred to the loss of carboxylic acid from the parent ion. The peak at *m*/*z* 137 represented the ion of hydroxybenzoic acid, which was formed by losing a sulfoxy group at the *o*-position of the phenol group from the parent ion. A further loss of a carboxylic acid group yielded a phenol ion with *m*/*z* 93. Based on the predicted molecular formula and the MS/MS fragmentation of compound **4**, we concluded that compound **4** is either 4-(sulfooxy)benzoic acid or its positional isomers (*o-*positional isomer, 2-(sulfooxy)benzoic acid; *p-*positional isomer, 3-(sulfooxy)benzoic acid). Another metabolite, compound **3**, with deprotonated molecular ion at *m*/*z* 232.9775 and at a retention time of 6.20 min, was identified as C_7_H_6_O_7_S. The MS/MS fragmentation pattern (Figure 5E) supported that the chemical structure of compound 3 is hydroxyl-(sufooxy)benzoic acid. The peak at *m*/*z* 189 referred to the loss of carboxylic acid from the parent ion. The peak at *m*/*z* 153 represented the ion of dihydroxybenzoic acid, which was formed by losing a sulfoxy group at the *o*-position of the hydroxyphenol group from the parent ion. A further loss of a carboxylic acid group yielded a benzene-diol ion with *m*/*z* 109.

**Figure A1:**
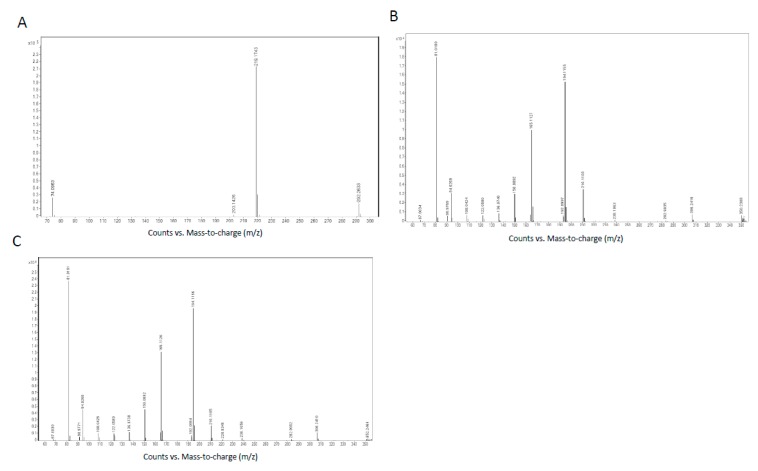
MS/MS spectra of metabolites with protonated molecular ions with (**A**) *m*/*z* 292.2645 eluted at 19.55 min; (**B**) *m*/*z* 352.2466 eluted at 28.5 min and (**C**) *m*/*z* 352.2466 eluted at 28.8 min.

The metabolite (compound **6** in Table 1), with [M + H]^+^ of *m*/*z* 292.2645, at a retention time of 19.55 min, and with molecular formula C_19_H_33_NO, matched 17β-amino-5α-androstan-11β-ol by database search in METLIN. However, the MS/MS fragmentation pattern was not compatible with the chemical structure of the matched compound (Figure A1). Similar discrepancies between the matched compound by database search and MS/MS fragmentation patterns were also observed in another two metabolites, compounds **7** and **8** in Table 1. These two metabolites, at retention time of 28.5 and 28.8 min, with the same [M + H]^+^ of *m*/*z* 352.2466 and molecular formula of C_20_H_33_NO_4_, were both matched to *N*-3-oxo-hexadec-11(*Z*)-enoyl-l-Homoserine lactone by database search in METLIN. However, the MS/MS fragmentation patterns of these two metabolites were not compatible with the chemical structures of the matched compounds (Figure A1). Since compound **7** and compound **8** had the same molecular formula, as well as their similar retention times and MS/MS spectra, these two compounds were proposed to be diastereomers. Compounds **6**–**8** listed in Table 1 are the potentially novel metabolites.

## 3. Discussion

In this study, by comparing the metabolomic profiles of the six *Aspergillus* species and 10 non-*Aspergillus* fungal species, we identified at least eight *Aspergillus* species-specific compounds in *Aspergillus* culture supernatants. The six *Aspergillus* species used were the four most commonly used *Aspergillus* species encountered in clinical specimens, including *A. fumigatus*, *A. flavus*, *A. niger* and *A. terreus*, as well as *A. nomius* and *A. tamarii*, two *Aspergillus* species commonly misidentified as *A. flavus* [17]. The control non-*Aspergillus* fungal species include commonly encountered yeasts (*Candida albicans* and *Cryptococcus neoformans*), molds (*Monascus pilosus*, *Phaeoacremonium parasiticum*, *Trichophyton rubrum*, *Trichophyton interdigitale* and *Lichtheimia* species) and thermal dimorphic fungus in our locality (*P. marneffei*). Multivariate analyses by unsupervised PCA and supervised PLS-DA showed that the *Aspergillus* species and non-*Aspergillus* species were clustered separately into two groups, indicating that there were potential metabolite(s) that could be used to differentiate them (Figure 2 and Figure 3). Subsequent extraction, statistical analysis and manual verification of the MFs revealed eight compounds that could be detected in the culture supernatants of all the six *Aspergillus* species but none of the other control fungal species. Since both the *Aspergillus* and non-*Aspergillus* species were cultured in the same culture medium, these compounds were genuine metabolites of the *Aspergillus* species. These *Aspergillus* specific fungal compounds and/or their metabolites may represent specific molecules that can be potentially detected in serum and/or urine of patients with invasive aspergillosis. Further studies using serum and urine samples of patients with culture and histology documented invasive aspergillosis should be performed to investigate which of these compounds and/or their metabolites could be detected in patients with aspergillosis for use in clinical microbiology laboratories.

One of the eight metabolites, compound **5** (Leu–Glu–Leu–Glu), observed in the six pathogenic *Aspergillus* species but not in other fungi, is a novel tetrapeptide specific to the *Aspergillus* species. Tetrapeptides are ribosomally or non-ribosomally synthesized secondary metabolites with biological functions. Ribosomally synthesized tetrapeptides are synthesized by ribosomal peptide synthetic (RiPS) pathway, whereas non-ribosomally synthesized tetrapeptides are synthesized by non-ribosomal peptide synthase (NRPS) gene clusters. Structurally, tetrapeptides can be linear or cyclic. In *Aspergillus* species, only two tetrapeptides are known (Table 2). The first one is ustiloxin B, a ribosomally synthesized cyclic tetrapeptide with sequence Tyr–Ala–Ile–Gly, the tyrosine residue of which is modified with norvaline, found in *A. flavus* [21]. The other one is asperterrestide A, a cyclic tetrapeptide with sequence anthranilic acid-3-OH-*N*-Me-Phe–Ile–Ala, found in *A. terreus* [22]. The tetrapeptide Leu–Glu–Leu–Glu discovered in the present study represents the first linear tetrapeptide observed in *Aspergillus* species, which we propose to be named aspergitide. We searched the genomes of *A. fumigatus*, *A. flavus*, *A. niger* and *A. terreus* but found no DNA sequence that potentially encodes repetitive Leu–Glu–Leu–Glu as observed in the ustiloxin B gene cluster in the *A. flavus* genome [21]. Therefore, we speculate that Leu–Glu–Leu–Glu is synthesized by NRPS cluster instead of RiPS pathway. Since the six *Aspergillus* species are members in the *Fumigati*, *Flavi*, *Nigri* and *Terrei* sections, Leu–Glu–Leu–Glu may be a tetrapeptide conserved across many sections in this genus; or alternatively, as the six *Aspergillus* species in this study are all pathogenic fungi, Leu–Glu–Leu–Glu could be a tetrapeptide with virulence properties.

**Table 2:** Known tetrapeptides in *Aspergillus* species.

Tetrapeptide	Sequence	Linear/Cyclic	Ribosomally/Non-Ribosomally Synthesized	*Aspergillus* Species from Which Tetrapeptide Is Found	Biological Properties
Ustiloxin B [21]	Tyr–Ala–Ile–Gly	Cyclic	Ribosomally synthesized	*A. flavus*	Mitotic inhibitor
Asperterrestide A [22]	^a^ ABA–3-OH-*N*-Me-Phe–Ile–Ala	Cyclic	Unknown	*A. terreus*	Inhibitory effects on influenza virus H1N1 and H3N2, cytotoxicity against human carcinoma cell lines
Aspergitide	Leu–Glu–Leu–Glu	Linear	Unknown	*A. flavus*, *A. niger*, *A. fumigatus*, *A. terreus*, *A. nomius*, *A. tamarii*	Unknown

^a^ ABA—Anthranilic acid.

**Figure 6:**
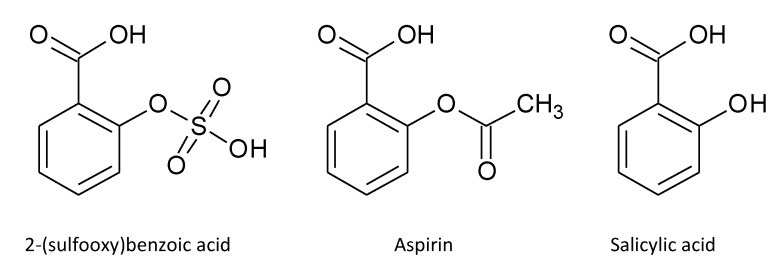
Chemical structures of 2-(sulfooxy)benzoic acid, aspirin and salicylic acid.

Two other metabolites, compound **3** [hydroxy-(sulfooxy)benzoic acid] and compound **4** [(sulfooxy)benzoic acid)], specific to the *Aspergillus* species may possess anti-inflammatory properties. 2-(sulfooxy)benzoic acid possesses a structure similar to those of aspirin [2-(acetoxy)benzoic acid] and salicylic acid (2-hydroxybenzoic acid) (Figure 6), which are potent analgesics and anti-inflammatory agents used for decades for treatment of various inflammatory conditions through inhibition of cyclooxygenases (COXs). Notably, it has been shown by molecular docking experiments, during the process of searching for analogues of gallic acid, a cyclooxygenase-2 (COX-2) inhibitor screened from medicinal plant, that 3-hydroxy-4-(sulfooxy)benzoic acid could be a potent COX-2 inhibitor [23]. However, both (sulfooxy)benzoic acid and hydroxy-(sulfooxy)benzoic acid have never been found naturally. In the present study, we showed that both of these compounds could be found in the metabolomes of the six pathogenic *Aspergillus* species. Since it has previously been reported that *o*-hydroxybenzoic acid and *p*-hydroxybenzoic acid were fungal secondary metabolites [24], we speculate that (sulfooxy)benzoic acid was formed by *o*-sulfation of hydroxybenzoic acid and hydroxy-(sulfooxy)benzoic acid was in turn formed by further oxidation of (sulfooxy)benzoic acid in the *Aspergillus* species. Further experiments will reveal whether these two metabolites of the pathogenic *Aspergillus* species are their virulent factors through suppression of the host inflammatory response.

## 4. Materials and Methods

### 4.1. Aspergillus Strains and Culture

Thirty *Aspergillus* strains of *A. fumigatus*, *A. flavus*, *A. niger*, *A. terreus*, *A. nomius* and *A. tamarii*; and 34 non-*Aspergillus* fungal strains, belonging to *C. neoformans*, *M. pilosus*, *C. albicans*, *P. parasiticum*, *P. marneffei*, *T. rubrum*, *T. interdigitale* and *Lichtheimia* species, were included in this study (Table 3). All clinical isolates were identified by standard phenotypic tests [25]. In addition, the identities of the *Aspergillus* strains were confirmed by ITS, β-tubulin and calmodulin gene sequencing [17]; *M. pilosus* and *Trichophyton* species were confirmed by ITS gene sequencing; *P. parasiticum* was confirmed by ITS and β-tubulin gene sequencing [26]; and those of the *Lichtheimia* species were confirmed by ITS, β-actin, EF1α and 28S rRNA gene sequencing [27,28]. All fungal strains were grown at a concentration of 2 × 10^6^ CFU in 10 mL RPMI 1640 medium (Gibco, Grand Island, NY, USA) supplemented with 2% glucose with shaking at 250 rpm. Strains were cultured for 48 h at 37 °C for *Aspergillus* species, *C. neoformans*, *M. pilosus*, *C. albicans*, *P. parasiticum* and *Lichtheimia* species, 108 h at 37 °C for *T. rubrum* and yeast form *of P. marneffei* and 108 h at 25 °C for mold form of *P. marneffei* to reach their early stationary phases to achieve comparable fungal concentrations. The culture supernatant was filtered using 0.22-μm MCE filter (Millipore, Billerica, MA, USA) and quenched immediately in liquid nitrogen for 10 min. The filtrates were lyophilized and stored at −80 °C until sample extraction and analysis. Uninoculated culture medium was used as a negative control. All strains were grown in duplicates.

**Table 3:** Fungal strains used in this study.

Fungal Species	Strains	Source of Strains
*Aspergillus fumigatus*	AF293, QC5096, PW1353, PW1354, PW1355, PW3460, PW3461, PW3462	AF293, purchased from ^a^ FGSC; QC5096, QC strain (specimen 5096) from ^b^ UKNEQAS; others, clinical isolates from Hong Kong
*Aspergillus flavus*	ATCC204304, PW2952, PW2953, PW2954, PW2956, PW2957, PW2960, PW2961, PW2962	ATCC204304, purchased from ^c^ ATCC; others, clinical isolates from Hong Kong [17]
*Aspergillus niger*	ATCC10577, PW3463, PW793, PW3464, PW3465	ATCC10577, purchased from ^c^ ATCC; others, clinical isolates from Hong Kong
*Aspergillus terreus*	PW3466, PW3467, PW3468	Clinical isolates from Hong Kong
*Aspergillus nomius*	PW2955, PW2959, CBS260.88	CBS260.88, purchased from ^d^ CBS Fungal Biodiversity Centre; others, clinical isolates from Hong Kong [17]
*Aspergillus tamarii*	PW2958, CBS104.13	PW2958, clinical isolate from Hong Kong [17]; CBS104.13 purchased from ^d^ CBS Fungal Biodiversity Centre
*Trichophyton rubrum*	PW3469	Clinical isolate from Hong Kong
*Trichophyton interdigitale*	PW3470, PW3471	Clinical isolates from Hong Kong
*Cryptococcus neoformans*	CBS132, PW275	PW275, clinical isolate from Hong Kong; CBS132, purchased from ^d^ CBS Fungal Biodiversity Centre
*Monascus pilosus*	PW2536	Clinical isolate from Hong Kong
*Phaeoacremonium parasiticum*	PW2367, PW2483	Clinical isolates from Hong Kong [26]
*Candida albicans*	ATCC90028, PW904, PW905, PW906	ATCC90028, purchased from ^c^ ATCC; others, clinical isolates from Hong Kong
*Penicillium marneffei*	PM1, PM35, PM41	Clinical isolates from Hong Kong [29]
*Lichtheimia corymbifera*	PW1473, PW1449, PW1471, HKU25, PW1450, PW1472	HKU25, QC strain (specimen 4727) from ^b^ UKNEQAS; others, clinical isolates from Hong Kong
*Lichtheimia ramosa*	PW2398, PW2399, HKU21, HKU22, HKU23, PW1493, PW1494, PW1495, PW1496	HKU23, QC strain (specimen 8273) from ^b^ UKNEQAS; others, clinical isolates from Hong Kong [27]
*Lichtheimia hyalospora*	CBS173.67	Purchased from ^d^ CBS Fungal Biodiversity Centre

^a^ FGSC—Fungal Genetics Stock Centre; ^b^ UKNEQAS—United Kingdom National External Quality Assessment Service; ^c^ ATCC—American Type Culture Collection; ^d^ CBS—Centraal Bureau voor Schimmelcultures.

### 4.2. Chemicals and Reagents

LC–MS grade water, methanol and acetonitrile were purchased from J.T. Baker (Center Valley, PA, USA). Formic Acid with ACS reagent grade was purchased from Sigma-Aldrich, Inc. (Saint Louis, MO, USA).

### 4.3. Sample Preparation

The samples were prepared according to our previous publications with minor modifications [17,19,30]. Lyophilized samples were reconstituted in 600 µL solvent mixture containing water/methanol/acetonitrile (2:4:4) (*v*/*v*/*v*). The sample mixtures were vortexed for 1 min followed by 10 min sonication at room temperature. After centrifugation at 14,000 rpm for 10 min at 4 °C, the supernatants were transferred to UHPLC–ESI-Q-TOF-MS analysis. To prevent batch effect, all specimens were analyzed in random manner.

### 4.4. UHPLC–ESI-Q-TOF-MS Analysis

UHPLC–ESI-Q-TOF-MS analysis was performed according to our previous publications with minor modifications [17,19,30]. For liquid chromatography, separations were performed using Agilent 1290 Infinity UHPLC (Agilent Technologies, Waldronn, Germany) and Waters Acquity UPLC HSS T3 (3.0 × 100 mm, 1.8 µm) column with Waters Acquity UPLC HSS T3 (2.1 × 5.0 mm, 1.8 µm) VanGuard Pre-column, The injection volume was 5 µL of each sample. The column and autosampler temperature were maintained at 45 and 15 °C respectively. Separation was performed at a flow rate of 0.4 mL/min under a gradient program. Mobile phase A was 0.1% formic acid (*v*/*v*) in water. Mobile phase B was pure methanol. The gradient program was applied as follows: *t* = 0 min, 1% B; *t* = 2.0 min, 1% B; *t* = 12 min, 38% B; *t* = 30 min, 99.5% B; *t* = 35 min, 99.5% B; *t* = 35.1 min, 1% B; *t* = 38 min, 1% B. Mass spectrometry was operated in both positive and negative ESI mode using Agilent 6540 Q-TOF mass spectrometer (Agilent Technologies, Santa Clara, CA, USA) with Agilent Jet Stream Electrospray ionization (ESI) source. The capillary voltage was kept at +3800 V with the nozzle voltage of +0 V in positive mode and −3500 V with the nozzle voltage of −0 V in negative mode. Other conditions were kept constant in all experiment. The gas temperature was kept at 320 °C. The drying gas (nitrogen) was set at 8 L/min. The pressure of the nebulizer gas (nitrogen) was maintained at 40 psi. The sheath gas was kept at a flow rate of 10 L/min at 380 °C. The voltages of the Fragmentor, Skimmer 1 and OctopoleRFPeak were kept at 135, 50 and 500 V, respectively. The scan range was adjusted to *m*/*z* 80–1700 at the acquisition rate of 2 spectra/s. Ultra-high purity nitrogen was used in product ion scanning (PIC) experiments. Product ion scanning was conducted using ultra-high purity nitrogen. MS/MS acquisition was operated in the same parameter as in MS acquisition. Collision Energy (CE) was set at 10, 20 or 40 eV for fragmentation of the targeted compounds to generate the best MS/MS spectra.

### 4.5. Data Processing and Statistical Data Analysis

The raw data from UHPLC–ESI-Q-TOF-MS and MS/MS were analyzed using Agilent Masshunter Qualitative Analysis software (version B.05.00, Agilent Technologies, Santa Clara, CA, USA). Multivariate analysis was used to examine the LC–MS data. Using molecular feature extraction (MFE) algorithm for automated baseline correction, noise calculation, peak detection and chromatogram deconvolution, molecular features (MFs) characterized by retention time (RT), chromatographic peak intensity and accurate mass were obtained. Data were converted into compound exchange file format (.cef) and analyzed using Mass Profiler Professional (MPP) software package (version B.02.02, Agilent Technologies, Santa Clara, CA, USA) for data filtering, peak alignment and statistical analysis. For the data filtering process, MFs with abundance either lower than 8000 counts per second (cps) or less than two isobaric mass peaks were removed. Alignment of RT and *m*/*z* values was performed across the sample sets with a tolerance window of ±0.2 min and ±10 mDa respectively. Data were normalized and baseline transformation was performed according to the median expression level of all data. For biomarker discovery and noise reduction, MFs having more than 50% occurrence either in *Aspergillus* strains or in non-*Aspergillus* strains were included for statistical analysis.

To identify the biomarkers in culture supernatant, univariate analysis was used. One-way analysis of variance (ANOVA) with Tukey’s *post-hoc* test was used to identify statistical significant biomarkers specifically present in the *Aspergillus* strains but not in non-*Aspergillus* fungal strains, where *p*-value <0.01 and fold-change >16 were considered as statistically significant. MFs were further filtered using Volcano Plot for *Aspergillus* and non-*Aspergillus* groups’ comparison. To reduce non-significant MFs, Student’s *t*-test with Benjamini-Hochberg correction was used, and MFs with *p*-value <0.01 were selected for further processing. MFs are sequentially filtered by fold-change (*FC*) analysis (*FC* > 16) to find MFs that have high abundance ratios between the two groups.

Stepwise reduction of the data dimensionality was followed by multivariate statistical analysis for PCA. PCA was performed by unsupervised pattern recognition technique. MFs specific to the *Aspergillus* strains were extracted from the PCA loading plot. PLS-DA was performed by supervised algorithm for the identification of important variables with discriminative power to explore the structure of the data and construct classification models. Box-and-whisker plots were generated using Analyse-it (Analyse-it Software, Leeds, UK). Abundance of metabolites specific to the *Aspergillus* strains with *p*-values <0.01, calculated by student’s *t*-test, was re-extracted from the raw data files of all the samples to re-confirm the metabolites were unique to the *Aspergillus* strains. The unique MFs of *Aspergillus* species were further studied for putative identification.

### 4.6. Metabolite Identification

Accurate masses of the unique MFs in *Apergillus* group were selected for product ion scanning. MS/MS spectra of the potential biomarkers were processed using Agilent MassHunter Qualitative Analysis software (version B.05.00, Agilent Technologies, Santa Clara, CA, USA) to generate potential molecular formula, based on the accurate mass and isotopic pattern recognitions of their parent and fragment ions. All putative biomarkers were confirmed by matching METLIN database (http://metlin.scripps.edu/), Human Metabolome Database (HMDB) (http://www.hmdb.ca/), MassBank (http://www.massbank.jp/), *E. coli* Metabolome Database (ECMDB) (http://www.ecmdb.ca/), LipidMaps (http://www.lipidmaps.org/), and KEGG database (http://www.genome.jp/kegg) search using exact molecular weights or MS/MS fragmentation pattern data and literature search. Efforts were made to distinguish metabolites from the other isobaric compounds whenever possible by their elution order and virtue of difference in fragmentation pattern corresponding to their structural characteristics.

## Appendix

**Table A1:** Median of the relative abundance of metabolites produced in *Aspergillus* species.

Metabolites	*A. flavus*	*A. fumigatus*	*A. niger*	*A. nomius*	*A. tamarii*	*A. terreus*
7-Methylguanine	17.772	33.947	27.289	13.295	17.071	2.750
1-Methylguanine	30.889	62.172	13.046	19.916	22.300	5.938
Hydroxy(sulfooxy)benzoic acid	5.244	1.059	1.379	1.866	42.364	8.141
(sulfooxy)benzoic acid	27.090	33.055	58.020	13.822	11.993	19.945
Leu–Glu–Leu–Glu	64.978	1.332	7.029	38.083	11.068	0.0145
Protonated molecular ion of *m*/*z* 292.3	9.885	4.691	25.409	5.392	18.912	15.520
Protonated molecular ion of *m*/*z* 352.2	9.035	3.567	36.472	8.992	12.176	24.447
Protonated molecular ion of *m*/*z* 352.2	3.965	2.126	11.142	4.273	5.556	9.046
